# Noninvasive electrical stimulation on pain and function in knee osteoarthritis in middle-aged and older adults: systematic review and meta-analysis of randomized clinical trials

**DOI:** 10.3389/fmed.2026.1792432

**Published:** 2026-04-10

**Authors:** Marta Cano-Orihuela, Agustín Aibar-Almazán, Paulino Vico-Rodríguez, Fidel Hita-Contreras, Yolanda Castellote-Caballero

**Affiliations:** 1 Department of Health Sciences, Faculty of Health Sciences, University of Atlántico Medio, Las Palmas de Gran Canaria, Spain; 2 Department of Health Sciences, Faculty of Health Sciences, University of Jaén, Jaén, Spain

**Keywords:** knee osteoarthritis, middle-aged, non-invasive electrical stimulation, older adults, pain, physical function

## Abstract

**Introduction:**

Knee osteoarthritis (KOA) is one of the most common musculoskeletal disorders in older adults, often leading to chronic pain, functional decline, and reduced quality of life. Conventional rehabilitation strategies frequently achieve only partial relief, which has stimulated interest in non-invasive electrical stimulation (NIES) as an adjunctive therapy. Therefore, the aim of this study was to synthesize the available evidence on the effects of NIES on pain and function in middle-aged and older adults with knee osteoarthritis.

**Methods:**

This systematic review and meta-analysis, conducted in accordance with PRISMA guidelines, evaluated randomized and controlled trials investigating the effects of transcranial direct current stimulation (tDCS), neuromuscular electrical stimulation (NMES and WB-EMS), transcutaneous electrical nerve stimulation (TENS), and innovative modalities such as cranial electrical stimulation (CES) and transcutaneous vagus nerve stimulation (tVNS).

**Results:**

A total of 15 randomized controlled trials, including 1,137 participants, met the inclusion criteria. The overall pooled analysis demonstrated significant improvements in pain and function with NIES, though heterogeneity was high. Subgroup analyses showed that tDCS produced consistent, moderate analgesic effects, while NMES and WB-EMS improved quadriceps strength, mobility, and patient-reported outcomes, especially in individuals with muscle weakness. TENS yielded the most variable results, with limited efficacy beyond short-term analgesia. CES and tVNS were supported by only two small trials, suggesting potential benefits but precluding pooled analysis.

**Conclusion:**

NIES appears to be a safe and promising adjunct in multimodal rehabilitation for KOA, though further large-scale, high-quality trials are required to confirm its clinical effectiveness.

**Systematic Review Registration:**

identifier: CRD420251174723.

## 1 Introduction

Knee osteoarthritis (KOA) is one of the leading causes of chronic pain, functional decline, and reduced quality of life in older adults, significantly increasing both the individual burden and the demand on healthcare systems (1, 2). The prevalence of KOA in this population is particularly high: it has been estimated that approximately 22.9% of individuals aged 40 years and older present with KOA, and that one in three adults over the age of 65 is affected by some form of osteoarthritis (3). Globally, around 7%−8% of the population is affected by osteoarthritis, with projections indicating a substantial increase in KOA by 2050 due to demographic aging (4). From a public health perspective, musculoskeletal disorders, including KOA, are among the leading causes of years lived with disability, restricting mobility and social participation in older age (5). In this context, older adults accumulate several risk factors that exacerbate disease severity, such as quadriceps weakness and associated neuromuscular alterations (6, 7), reduced physiological recovery capacity associated with aging (8), high prevalence of comorbidities (9, 10), neuromuscular alterations (6, 7), and an increased susceptibility to adverse pharmacological effects, particularly from prolonged use of non-steroidal anti-inflammatory drugs (NSAIDs) (11). These factors highlight the need for tailored and safe treatment strategies for KOA in the older population.

One of the key alterations in KOA is the involvement of the quadriceps femoris muscle, whose weakness has been linked to increased pain, progression of joint degeneration, knee instability, and a worse functional prognosis (6, 12). In older adults with KOA, quadriceps atrophy, atherogenic neuromuscular inhibition, and decreased voluntary activation constitute significant barriers to recovery (13, 14). Weakness in this muscle group has also been identified as a predictor of mobility limitations and a higher risk of disability in this population (15, 16). Furthermore, neuromuscular dysfunction not only contributes to pain but also to impaired proprioception and balance, which increases the risk of falls and loss of independence in older adults (8, 17). This suggests that addressing periarticular musculature—in addition to traditional pain management—may play a key role in preserving function and slowing disability progression in older patients with KOA (7).

Among non-pharmacological intervention strategies, NIES, including neuromuscular electrical stimulation (NMES) and transcutaneous electrical nerve stimulation (TENS), has been proposed as an alternative or complement to conventional exercise in older adults with KOA (18, 19). Its appeal lies in the fact that it allows direct activation of muscle fibers or motor nerves, generating contractions or sensory modulations in a controlled manner, and thus overcoming some of the barriers that traditional exercise encounters in this population, such as pain at the beginning of the activity, limited load capacity, or the presence of comorbidities (20, 21). Several studies have shown that NMES applied to older adults with KOA produces improvements in quadriceps isometric strength and muscle structural parameters (13, 18). Along these lines, a systematic review and meta-analysis concluded that there is moderate evidence that NMES, alone or in combination with exercise, increases muscle strength and function in people with KOA (22). However, despite these encouraging results, the literature has important limitations: methodological heterogeneity, variability in intensity, electrode placement, duration and frequency of protocols, and the lack of trials specifically designed in the older population, which makes it difficult to extrapolate the findings to this age group (18, 22).

Furthermore, the issue of pain and function associated with KOA in older adults poses particular challenges. Improving muscle strength is not enough: in this population, it is essential to consider how the intervention impacts joint pain, the ability to walk, stand, climb stairs, and perform activities of daily living (23). Regarding the use of electrical stimulation for pain relief, a meta-analysis in patients with KOA that included different modalities (high- andv low-frequency transcutaneous stimulation, interferential current, NMES, among others) concluded that, although some techniques, particularly interferential current, showed promising results, methodological heterogeneity and variable study quality limit the strength of the available evidence (24). Thus, although electrical stimulation represents a potential alternative for pain control, its specific effectiveness in older adults with KOA is not yet clearly established and requires more focused studies in this population (25).

In this sense, the older adult population requires specific attention, since the presence of comorbidities, lower muscle reserve, higher risk of pharmacological adverse effects, low tolerance to physical exertion, and high interindividual variability in function limit the extrapolation of the results observed in younger populations (3, 4). The safety of electrotherapy interventions in people with KOA and multiple comorbidities has been explored, showing that these techniques do not produce clinically relevant increases in heart rate or the appearance of arrhythmias, which supports their feasibility in this age group (26). Even so, there is a persistent need to clarify the true magnitude of the effect of NIES on pain, muscle strength, and functionality in older adults with KOA. which protocols are most appropriate in terms of intensity, location, duration, and frequency; how factors such as previous muscle weakness, degree of osteoarthritis, comorbidities, baseline functional level, and treatment adherence influence them; and what the effects are on clinically relevant outcomes such as gait, chair-rising, stair-climbing ability, and health-related quality of life (24, 25).

Although several systematic reviews and meta-analyses have examined the effects of electrical stimulation in knee osteoarthritis, important limitations remain in the existing literature. Most previous syntheses have focused on single stimulation modalities, have combined invasive and non-invasive techniques, or have included broad age ranges without specific emphasis on middle-aged and older adults. In addition, methodological heterogeneity and inconsistent reporting of patient-reported outcomes and functional measures have limited definitive conclusions about clinical effectiveness (e.g., NMES combined with exercise shows inconsistent functional benefits) (27). Prior work on TENS and related peripheral stimulation has similarly reported variable results for physical function despite some beneficial effects on pain and walking ability (28). Moreover, network meta-analyses comparing multiple electrical stimulation modalities have highlighted the limited number of high-quality comparative trials and the challenges in interpreting pooled estimates across heterogeneous protocols (29). Addressing these gaps is essential to better understand the clinical relevance of NIES and to inform rehabilitation strategies aimed at preserving independence and functional capacity in older adults with knee osteoarthritis.

Importantly, the term non-invasive electrical stimulation (NIES) encompasses several modalities that operate through distinct physiological mechanisms and therapeutic targets. Some techniques primarily act through central neuromodulation mechanisms. For example, transcranial direct current stimulation (tDCS) and cranial electrical stimulation (CES) aim to modulate cortical excitability and influence descending pain inhibitory pathways. In contrast, transcutaneous electrical nerve stimulation (TENS) primarily produces peripheral sensory analgesia through activation of afferent Aβ fibers and modulation of nociceptive transmission at the spinal level. Neuromuscular electrical stimulation (NMES) and whole-body electromyostimulation (WB-EMS), on the other hand, are designed to activate motor units and induce muscle contractions, thereby improving muscle strength, neuromuscular control, and functional capacity. Recognizing these different mechanisms is important when interpreting clinical outcomes, as modalities targeting central pain processing may show stronger effects on pain reduction, whereas neuromuscular stimulation techniques are more closely associated with improvements in muscle strength and functional performance.

This systematic review and meta-analysis aim to synthesize the available evidence on the effects of NIES on pain and physical function in middle-aged and older adults with KOA. Specifically, this study was designed to address the following research questions: (1) what are the effects of NIES on pain and physical function in middle-aged and older adults with KOA compared with control or usual care interventions; (2) whether different NIES modalities, including transcranial direct current stimulation, neuromuscular and whole-body electromyostimulation, transcutaneous electrical nerve stimulation, and other emerging techniques, differ in their effects on pain and functional outcomes in this population; and (3) which characteristics related to the intervention or the participants appear to be associated with greater improvements in pain and physical function. The remainder of the article is organized as follows: Section 2 describes the materials and methods, Section 3 presents the results, Section 4 discusses the findings in the context of the existing literature, and Section 5 summarizes the main conclusions.

## 2 Materials and methods

This systematic review and meta-analysis was conducted following the recommendations of the 2020 PRISMA declaration (30), ensuring a transparent and standardized process for selecting and synthesizing available evidence. Furthermore, the methodological procedure was aligned with the guidelines contained in the Cochrane Handbook for Systematic Reviews of Interventions (31). The study protocol was previously registered in the international PROSPERO database under reference number CRD420251174723.

### 2.1 Information sources and search strategy

A comprehensive bibliographic search was conducted in four major biomedical databases: PubMed/MEDLINE, Scopus, Web of Science, and CINAHL. The electronic search was conducted from database inception until February 28, 2025. The search strategy was built by combining terms related to three main areas: (i) Knee osteoarthritis (e.g.,”knee osteoarthritis,” ”gonarthrosis‘'); (ii) NIES techniques (e.g., “transcutaneous electrical nerve stimulation,” “TENS,” “neuromuscular electrical stimulation,” “NMES,” “transcranial direct current stimulation,” “tDCS,” “vagus nerve stimulation,” “tVNS”); and (iii) Population (e.g., “older adults,” “elderly,” “middle-aged”). These descriptors were adapted to the controlled vocabulary of each database (MeSH in PubMed) and combined using Boolean operators (AND/OR). In addition, a manual search was performed in the reference lists of the included studies to identify additional eligible trials. Specifically, the PubMed search string used was: (“Osteoarthritis, Knee”[Mesh] OR “knee osteoarthritis”[tiab] OR gonarthrosis[tiab]) AND (“Transcutaneous Electric Nerve Stimulation”[Mesh] OR “transcutaneous electrical nerve stimulation”[tiab] OR TENS[tiab] OR “Neuromuscular Electrical Stimulation”[Mesh] OR “neuromuscular electrical stimulation”[tiab] OR NMES[tiab] OR “Transcranial Direct Current Stimulation”[Mesh] OR “transcranial direct current stimulation”[tiab] OR tDCS[tiab] OR “Vagus Nerve Stimulation”[Mesh] OR “vagus nerve stimulation”[tiab] OR tVNS[tiab]) AND (“Aged”[Mesh] OR “Aged, 80 and over”[Mesh] OR “Middle Aged”[Mesh] OR older adult^*^[tiab] OR elderly[tiab] OR “middle-aged”[tiab]) AND (random^*^[tiab] OR “Randomized Controlled Trial [Publication Type] OR ”controlled clinical trial“[Publication Type]). No language restrictions were applied during the search process. In addition to database searching, the reference lists of all included studies and relevant reviews were manually screened to identify additional eligible trials. Grey literature sources and clinical trial registries (e.g., ClinicalTrials.gov) were not systematically searched.

### 2.2 Selection criteria

Studies that met the following criteria were considered eligible: (i) Randomized controlled trials (RCTs) published in peer-reviewed journals; (ii) Population comprised of older adults with a clinical or radiological diagnosis of KOA, according to internationally accepted criteria; (iii) Intervention based on noninvasive electrical stimulation, including NMES, TENS, tDCS, or VNS; (iv) Presence of a comparator group (placebo, sham, treatment as usual, or conventional physical therapy/exercise interventions); (v) Reporting of at least one clinically relevant outcome (pain, muscle strength, physical function, gait ability, activities of daily living, or health-related quality of life). Studies in which NIES was combined with other interventions, such as therapeutic exercise or conventional physiotherapy, were eligible for inclusion provided that a comparator group was present and the effect of the electrical stimulation intervention could be reasonably isolated at the group level. When combined interventions were included, analyses were conducted according to the assigned intervention groups as reported in the original trials, and subgroup analyses were used to explore potential differences across stimulation modalities. Studies were excluded if they: (i) did not include a non-invasive electrical stimulation intervention or did not allow identification of the specific contribution of electrical stimulation; (ii) were conducted in populations without a confirmed diagnosis of knee osteoarthritis or in non-adult populations; or (iii) did not provide sufficient data for qualitative or quantitative synthesis.

### 2.3 Data extraction and reliability

The information from the included studies was collected using an extraction form designed specifically for this review. This form included relevant data such as author and year of publication, country, sample size and participant characteristics, type of intervention applied (stimulation modality, frequency, duration, and delivery format), comparator conditions, outcomes analyzed (pain, muscle strength, physical function, and quality of life), measurement instruments used, and main findings. Data extraction was performed independently by two reviewers with experience in systematic reviews and meta-analyses in the field of physical therapy and rehabilitation, in order to ensure consistency and minimize the risk of errors. Any discrepancies between reviewers were resolved through discussion and consensus, and when agreement could not be reached, a third researcher with extensive experience in evidence synthesis was consulted to arbitrate and make the final decision.

### 2.4 Assessment of methodological quality

The methodological rigor of the included trials was assessed using two complementary approaches with distinct purposes. In accordance with the Cochrane Handbook for Systematic Reviews of Interventions, the Cochrane Risk of Bias 2 (RoB 2) tool was used as the primary framework to assess the internal validity of randomized controlled trials (32). The RoB 2 tool evaluates five domains: the randomization process, deviations from intended interventions, missing outcome data, measurement of the outcome, and selection of the reported result. In parallel, the Physiotherapy Evidence Database (PEDro) scale was applied to provide an additional characterization of methodological quality and reporting features commonly used in physical therapy and rehabilitation research (33). The PEDro scale consists of 11 items, 10 of which contribute to the total score, as the first item (eligibility criteria) is reported separately. Each item is scored dichotomously (“Yes” = 1; “No” = 0), yielding a total score ranging from 0 to 10. For descriptive purposes, studies were classified as low (0–3), fair (4–5), good (6–8), or excellent quality (9–10). Importantly, PEDro scores were not used to infer risk of bias but rather to complement the RoB 2 assessment by summarizing key design and reporting characteristics. Both RoB 2 and PEDro assessments were performed independently by two reviewers with experience in systematic reviews and electrotherapy interventions, and all included studies were evaluated using both tools. Reviewers were not blinded to study authors or journals, which is consistent with standard practice in systematic reviews. Any disagreements were resolved through discussion and consensus, and when necessary, a third senior reviewer was consulted to arbitrate. Given the qualitative and domain-based nature of RoB 2 judgments, inter-rater reliability statistics (e.g., kappa coefficients) were not calculated. Although several studies were judged to have a low risk of bias in specific RoB 2 domains, particularly deviations from intended interventions, these judgments should be interpreted with caution. In electrical stimulation trials, participant blinding is inherently challenging, and residual performance bias cannot be fully excluded even when sham-controlled designs are used.

### 2.5 Data synthesis and analytical decisions for the meta-analysis

The results of the meta-analysis were represented using forest plots, detailing for each study the first author, year of publication, sample size, and estimated effect size (Hedges' g), along with its 95% confidence interval and corresponding *p*-value. This graphical representation facilitated the interpretation of both the magnitude of the effects and their statistical significance. To check the robustness of the synthesis, sensitivity analyses were performed by sequentially excluding studies with overlapping samples, outliers, or extreme estimates that could influence the pooled results. Comparison between the reduced models and the full analysis confirmed the stability of the overall effects. Subgroup analyses were also performed according to the nature of the outcome variables. Pain outcomes were assessed using validated patient-reported measures, including the Visual Analogue Scale (VAS), the pain subscales of the Western Ontario and McMaster Universities Osteoarthritis Index (WOMAC), the Knee injury and Osteoarthritis Outcome Score (KOOS), and the Brief Pain Inventory (BPI), as reported in the original trials. Physical function outcomes included both patient-reported measures (e.g., WOMAC and KOOS function subscales) and performance-based tests such as the Timed Up and Go test, stair-climbing tests, chair-stand tests, and the 6-min Walk Test. To allow pooling across studies using different instruments, outcomes were standardized using standardized mean differences. All outcomes were analyzed in the post-intervention period reported by the original trials. Due to the anticipated clinical and methodological heterogeneity among the studies, a random-effects model was used, which allowed for the incorporation of real variations between effect sizes and improved the generalizability of the findings. Between-study variance (τ^2^) was estimated using the DerSimonian–Laird method, and pooled effect sizes were calculated accordingly. Statistical heterogeneity was assessed using Cochran's Q test and the I^2^ statistic (31), with values greater than 50% being interpreted as indicating moderate or high heterogeneity, possibly derived from differences in methods, settings, or characteristics of the included populations. Finally, publication bias was explored using funnel plots, which allow for the visualization of potential asymmetries related to the tendency to publish positive or statistically significant results. This graphical assessment was complemented by Egger's test, used to statistically detect the presence of bias in the set of studies analyzed.

## 3 Results

### 3.1 Study selection process

**Figure 1:**
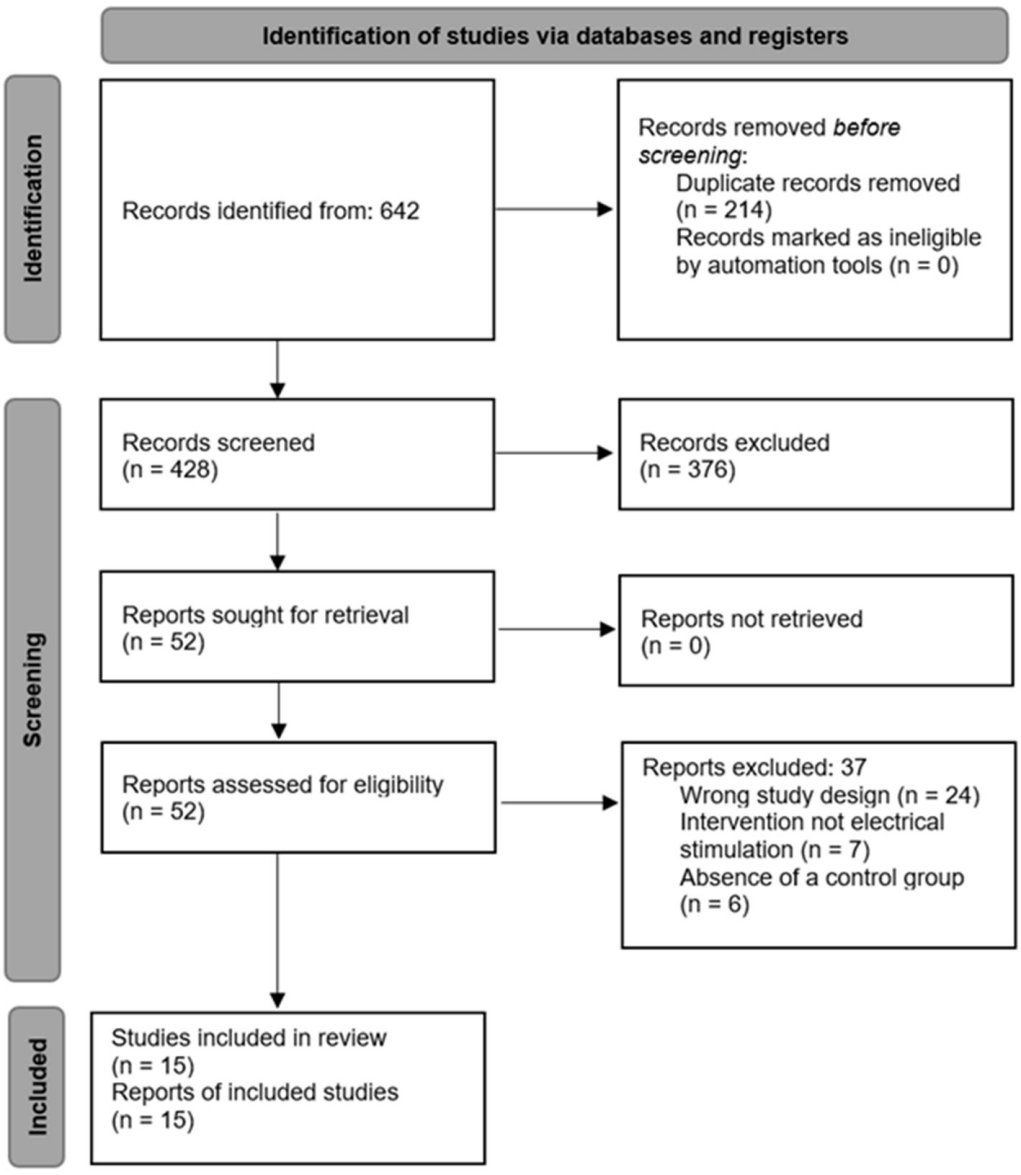
Study selection process flow chart.

The systematic search of the four databases (PubMed, Scopus, Web of Science, and CINAHL) identified a total of 642 records. After removing duplicates, 428 unique references remained for initial evaluation. Based on titles and abstracts, articles not meeting the predefined inclusion criteria were excluded, leaving 52 potentially relevant studies for full-text review. After detailed assessment, 15 randomized clinical trials fulfilled all eligibility criteria. The main reasons for exclusion at this stage were a study population not matching older adults, interventions not based on electrical stimulation, absence of a control group, outcomes unrelated to the study objective, or methodological limitations that prevented data extraction. The complete flow of the selection process is presented in Figure 1, in accordance with the PRISMA 2020 guidelines.

### 3.2 Methodological quality

The methodological quality of the 15 included trials was assessed using the PEDro scale. Reported scores ranged from 6 to 9 out of 10, indicating an overall moderate to high level of methodological rigor. No study achieved the maximum score, but five trials and reached nine points, and several others obtained scores of eight. The lowest scores were six. Most trials (about two-thirds) scored seven or higher, reflecting adequate reporting of randomization procedures, between-group comparisons, and outcome measures. Nevertheless, some methodological weaknesses were consistent across studies. The most frequent shortcomings were related to the lack of blinding of participants, therapists, or assessors, as well as limited description of allocation concealment. Overall, the quality of evidence can be considered satisfactory, but these gaps suggest a persistent risk of bias that should be taken into account when interpreting the results. Future research should prioritize more rigorous blinding procedures and detailed reporting of randomization to further strengthen internal validity. A detailed summary of the quality assessment is provided in Table 1.

**Table 1:** Methodological quality of the included articles.

Study	1	2	3	4	5	6	7	8	9	10	11	Total Score
Rahimi et al. (34)	0	1	0	1	1	0	1	1	0	1	1	7/10
Suchting et al. (42)	1	1	0	1	1	0	1	1	0	1	1	7/10
Suchting et al. (47)	1	1	1	1	0	0	0	1	0	1	1	7/10
Li et al. (46)	1	1	0	1	0	0	0	1	0	1	1	6/10
Tavares et al. (35)	1	1	1	1	1	0	1	1	1	1	1	9/10
Dasa et al. (44)	1	1	1	1	1	0	1	1	1	1	1	9/10
Sajadi et al. (36)	1	1	0	1	1	0	1	1	0	1	1	8/10
Ahn et al. (37)	1	1	1	1	1	0	1	1	0	1	1	8/10
Xia et al. (38)	1	1	1	1	1	0	1	1	0	1	1	8/10
Moezy et al. (39)	1	1	0	1	1	0	1	1	0	1	1	7/10
Kast et al. (40)	1	1	1	1	0	0	1	1	1	1	1	8/10
Iijima et al. (43)	1	1	1	1	1	0	1	1	0	1	1	8/10
Klika, et al. (45)	1	1	1	1	0	0	1	1	0	1	1	7/10
Reichenbach et al. (48)	1	1	1	1	1	0	1	1	1	1	1	9/10
Elsehrawy et al. (41)	1	1	1	1	1	0	1	1	1	1	1	9/10

Items: 1: eligibility criteria; 2: random allocation; 3: concealed allocation; 4: baseline comparability; 5: blind subjects; 6: blind therapists; 7: blind assessors; 8: adequate follow-up; 9: intention-to-treat analysis; 10: between-group comparisons; 11: point estimates and variability; yes = 1; no = 0.

**Figure 2:**
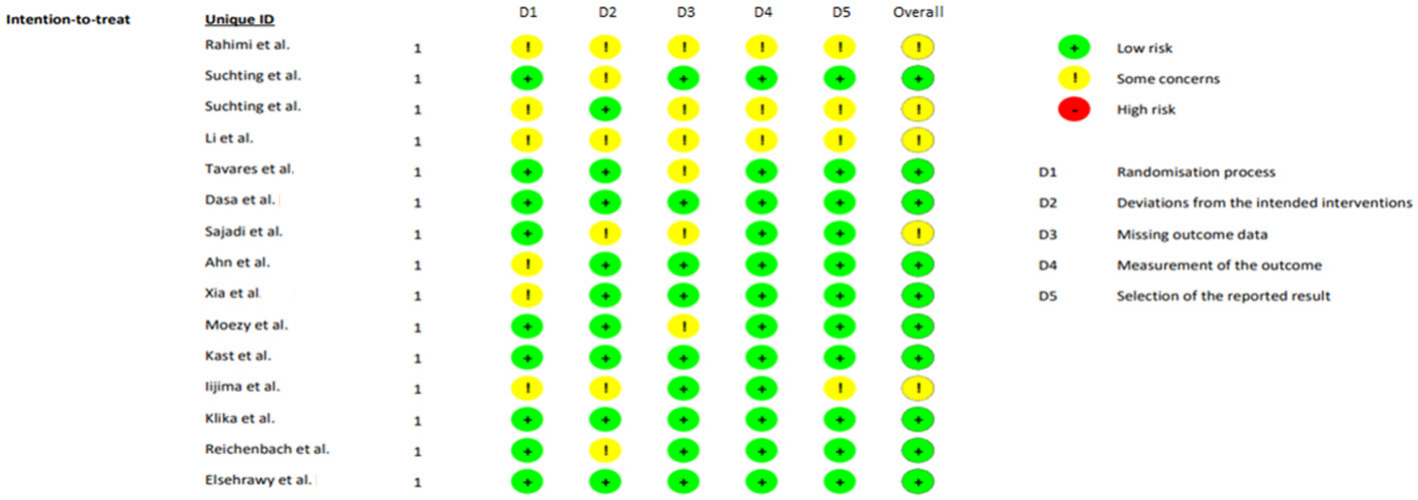
Risk of bias of the included articles.

Regarding the risk of bias assessment using the Cochrane Collaboration's ROB 2.0 tool, no trials at high risk of bias were identified in the set of studies assessed. Studies with a low overall risk predominated, along with a smaller number rated with some concerns, suggesting moderate-high overall confidence in the synthesized results. At the domain level, the main concerns were concentrated in D1 (randomization) and D2 (intervention biases), generally due to incomplete descriptions of the sequence/allocation concealment or blinding limitations that could have introduced post-randomization biases. In contrast, D3–D5 showed low risk in most studies, with manageable and balanced follow-up losses, use of valid outcome measures, and consistency between the analyses presented and the predefined results. Overall, the observed risk profile supports the robustness of the conclusions, although the “some concerns” in D1–D2 recommend cautious interpretation of small effects (Figure 2).

### 3.3 Characteristics of the studies

This systematic review comprised 15 randomized controlled trials published between 2020 and 2025, conducted in diverse geographic settings including the United States, Brazil, China, Iran, Japan, Switzerland, Germany, and Egypt. The included participants were predominantly older adults with KOA, with mean ages ranging from 47 to 74 years. Sample sizes varied notably, from 20 to 220 participants, with most studies including comparable numbers in the intervention and control groups. The interventions evaluated covered different forms of NIES. The most frequently investigated modality was tDCS, alone or combined with conventional physiotherapy, exercise, or other techniques such as electroacupuncture. Several trials focused on transcutaneous electrical nerve stimulation (TENS), while others examined NMES, including wearable or home-based protocols, and whole-body electromyostimulation (WB-EMS). One study evaluated tVNS as an innovative approach. Protocols showed high variability in terms of frequency, intensity, and duration. Session lengths ranged from 15 to 60 min, delivered between 2 and 26 weeks, with some interventions extending up to 7 months. Control conditions included sham stimulation, usual care, multimodal analgesia, or therapeutic exercise programs. Outcome measures were equally heterogeneous. The most frequently reported endpoints included pain intensity (measured with VAS, WOMAC pain, KOOS pain, or BPI), physical function (Timed Up and Go, stair-climbing, 6-min Walk Test, chair stand), and muscle strength (isometric quadriceps strength, ultrasound-based muscle thickness). Other outcomes comprised quality of life indices (SF-36, VR-12, PedsQL), inflammatory and stress biomarkers (IL-6, CRP, cortisol), and central sensitization or neurophysiological parameters (QST, fNIRS). Overall, despite notable methodological and clinical heterogeneity across trials, the studies consistently targeted pain reduction, functional improvement, and enhanced quality of life in older adults with KOA. Collectively, they provide an updated overview of the therapeutic potential of non-invasive electrical stimulation in this population (Table 2).

**Table 2:** Characteristics of the studies (*n* = 15).

Authors (year, country)	Study design	Mean age	Sample size (exercise/ control)	Control group	Type of intervention	Frequency and stimulation parameters	Session duration and total program time (weeks)	Outcomes and measures	Result
Rahimi et al. (34)	RCT	58.8 ± 3.3	80 (20/20)	PT + sham tDCS	PT + tDCS (M1, S1 o DLPFC) + PT	1 mA, 20 min, electrodes 4 × 4 cm, 10 sessions (5/week)	20 min tDCS + ~60 min PT per session; total 2 weeks (10 sessions)	VAS (pain), KOOS, quadriceps strength, ROM, stepping test (15 s), 30-s chair stand, 10-m walk test	Active tDCS, especially over M1, significantly improved pain, disability (KOOS), and functional performance vs. sham, with effects persisting at 1-month follow-up
Suchting et al. (42)	RCT	59.9 (9.2)	40 (20/20)	Sham tDCS	Active tDCS (M1-SO montage)	2 mA, 20 min, saline-soaked sponge electrodes (35 cm^2^), 5 sessions (1/day, 5 consecutive days)	20 min/session, 1 week (5 sessions total)	Blood biomarkers: IL-6, IL-10, TNF-α, CRP, cortisol, β-endorphin	Active tDCS significantly reduced inflammatory cytokines (IL-6, IL-10, TNF-α) and β-endorphin vs. sham. No significant changes in CRP or cortisol. Provides preliminary evidence of anti-inflammatory effects of tDCS
Suchting et al. (47)	RCT	61.2 ± 7.2	29 (17/12)	Sham tDCS	Home-based, remotely supervised tDCS	2 mA constant current; anode over M1, cathode supraorbital; saline-soaked sponge electrodes (5 × 7 cm)	20 minutes per session, 5 sessions per week, for 2 weeks (10 sessions total)	Quantitative Sensory Testing (heat pain threshold/tolerance, pressure pain threshold, punctate mechanical pain, conditioned pain modulation), VAS pain, WOMAC	Significant improvements in 7/11 QST measures (e.g., PPT medial and lateral knee, trapezius, CPM); longitudinal models supported improvements in 10/11 measures. Preliminary evidence of reduced pain sensitivity.
Li et al. (46)	RCT	66.1 ± 5.8	80 (40/40)	Standard multimodal analgesia (epidural + systemic drugs + rehab)	tDCS (M1 anodal, 1.5 mA, 20 min) + electroacupuncture (6 acupoints, 2/100 Hz, 2–5 mA, 30 min) in addition to standard care	tDCS: 5 × /week, 6 weeks (30 sessions); EA: daily, 6 weeks (42 sessions)	20–30 min per session; total 6 weeks	Pain: VAS at rest and movement; Function: KOOS, HSS score, knee flexion ROM; QOL: SF-36; Rehab outcomes: time to ambulation, hospital stay	Combined tDCS + EA significantly reduced pain (VAS), improved knee function (KOOS, HSS, ROM), enhanced quality of life (SF-36), shortened hospital stay, with no adverse effects compared to control
Brandão Tavares et al. (35)	RCT	73.9 ± 7.9	104 (51/53)	Sham tDCS	Active tDCS over primary M1 with cathode over contralateral SO	2 mA, 20 minutes/session, anode on M1 contralateral to most affected knee, cathode on SO contralateral	15 daily sessions (Mon–Fri), 3 weeks total	Brief Pain Inventory (BPI, pain intensity). VAS pain, WOMAC, Lequesne Index, Timed Up and Go Test (TUGT), One Leg Stance (OLS), health-related QoL (SF-12), conditioned pain modulation (CPM), pain pressure threshold (PPT), quantitative sensory testing	Active tDCS significantly reduced pain intensity (BPI mean reduction 2.88 vs. 1.29 in sham; Cohen's d = 0.58) and improved CPM. Effects were not maintained at 2-month follow-up. No serious adverse events.
Dasa et al. (44)	RCT	62.1 ± 9.93	64 (42/22)	Sham NMES	Home-based mobile app–guided NMES (CyMedica system; electrodes over quadriceps via knee garment)	Two 20-min sessions/day, 5 days/week; adjustable intensity up to max tolerable (0.5–16.4 V)	14 weeks (extension study, total 26 weeks with parent trial)	Pain (VAS for nominated activity, general VAS), WOMAC (pain, stiffness, function, total), isometric quadriceps strength, Timed Up & Go, 30-s chair rise test, PGIC, satisfaction	In ITT analysis: no significant between-group differences. In per-protocol compliant group: active NMES led to significantly greater reductions in VAS activity pain (−64.7 vs. −24.3%), improved WOMAC function and total scores, and higher responder rates (>50% pain reduction). Quadriceps strength increased significantly in the active NMES group. 95% reported moderate-to-high satisfaction.
Sajadi et al. (36)	RCT	56.8 ± 5.81	40 (20/20)	TENS (conventional: 100 Hz, 100 μs, 10% below motor threshold, 25 min, 6 sessions in 2 weeks)	TENS: conventional TENS applied to painful knee area (100 Hz, 100 μs, intensity 10% below motor threshold). tDCS: anodal stimulation of M1 (C3/C4 contralateral to affected knee), cathode over ipsilateral supraorbital area; 2 mA. Both groups also performed knee strengthening exercises twice daily.	TENS: 100 Hz, 100 μs pulse width, 25 min/session. tDCS: 2 mA constant current, 20 min/session.	3 sessions/week for 2 weeks → 6 sessions total	Visual Analogue Scale (VAS); WOMAC (pain, stiffness, function). Assessments at baseline, 1 week, 1 month, and 3 months after intervention.	Both groups improved significantly in pain and function. No significant difference between TENS and tDCS. Improvements sustained up to 3 months. No adverse events.
Ahn et al. (37)	RCT	59.43 ± 6.27	30 (15/15)	Sham CES	Active CES, remotely supervised via secure videoconferencing, Alpha-Stim M device	0.1 mA, 0.5 Hz, 60 min/day, 5 days/week	10 sessions over 2 weeks	Primary: Numeric Rating Scale (NRS) for knee pain. Secondary: Heat pain threshold/tolerance, pressure pain threshold (PPT), conditioned pain modulation (CPM), fNIRS cortical response, Client Satisfaction Questionnaire (CSQ-8)	Active CES significantly reduced NRS pain (*d* = 1.43), increased heat pain threshold (*d* = 1.40), PPT (*d* = 1.50), and CPM (*d* = 1.95) vs. sham. fNIRS showed reduced frontal cortical hemodynamic activity. High feasibility and acceptability, no adverse effects.
Xia et al. (38)	RCT	64.96 ± 2.77	110 (55/55)	Wearable TENS + sham tDCS	Wearable TENS + active tDCS (anodal Cz, cathodal supraorbital)	TENS: 1–250 Hz sweep, 60 ms, intensity ~70% motor threshold, 30 min/day, 5 × /week, 4 weeks (20 sessions); tDCS: 2 mA, 20 min, 5 × /week, 4 weeks (20 sessions)	20–30 min/session, 4 weeks	Primary: brief Pain Inventory (BPI). Secondary: VAS pain, step length, cadence, 6MWT, active knee ROM, quadriceps strength	TENS + tDCS significantly reduced pain (BPI, VAS) and improved gait parameters (step length, 6MWT) compared to TENS + sham. Effects sustained at 1 month post-treatment but not at 2 months.
Moezy et al. (39)	RCT	57.31 ± 5.80	75 (25/25/25)	Exercise therapy alone (isometric quadriceps, straight leg raise, terminal knee extension, hip adductor isometrics, wall-sit)	NMES alone (medium-frequency, interferential premodeled current 4,000–4,050 Hz, beat frequency 50 Hz, 600 μs pulse width, electrodes over rectus femoris and VMO, intensity adjusted to visible contraction); NMES + exercise	12 supervised sessions, 3 × /week for 4 weeks.	Cada sesión ~15 min de NMES + ejercicios (30–40 min). Total: 4 semanas (12 sesiones).	Pain (VAS), FROM knee flexion, thigh girth (TG), VMO thickness (ultrasound), Timed Up and Go (TUG), 6MWT, WOMAC (pain, stiffness, function)	NMES group: greatest reduction in pain and mobility (TUG, 6MWT). NMES + exercise: best outcomes in ROM, muscle thickness, WOMAC stiffness/function. Exercise alone: less effective overall. Benefits persisted at 12 weeks.
Kast et al. (40)	RCT	57.9 ± 7.0	72 (36/36)	Usual care group (6 physiotherapy sessions over 3 months + education program)	Whole-body electromyostimulation (WB-EMS)	Bipolar current, 85 Hz, 350 μs impulse width; 6 s stimulation/4 s rest cycles; stimulation of up to 10 muscle groups with suit electrodes; intensity regulated by RPE (6–7 CR10)	20 min per session, 3 times per fortnight, over 7 months (including 1 month conditioning)	Primary: KOOS-Pain. Secondary: KOOS subscales (symptoms, ADL, sport/rec, QoL), 7-day pain diary (NRS), hip/leg extensor strength (isokinetic leg press), 30-s sit-to-stand test, body composition, analgesic use	Significant between-group improvements in KOOS-Pain (+18.2%, SMD=0.65, *p* = 0.004) and all other KOOS subscales (*SMD* = 0.62–0.78). Greater reduction in pain intensity (NRS MD −1.04, *p* = 0.005), increase in hip/leg strength (+79 N, *p* = 0.03), and sit-to-stand repetitions (+3.9, *p* < 0.001). Reduced analgesic use in WB-EMS group. No adverse events reported.
Iijima et al. (43)	RCT	59.9 ± 6.41	59 (30/29)	Sham TENS	TENS applied to the index knee	Prototype TENS device (HV-F710, Omron); sweep mode 1–250 Hz, symmetrical biphasic pulse, 60 μs pulse width; intensity adjusted to “strong but comfortable, non-painful tingle” below motor threshold	~60 min, single-session application (assessed immediately post-treatment)	11-step stair climb test (SCT, stopwatch and insole-based); VAS for knee pain during SCT; quadriceps muscle strength	TENS significantly improved insole-based SCT time by 0.41 s (95% CI −0.75 to −0.07, *p* < 0.05) vs. sham. Effect mainly due to reduced transition phase time (−0.32 s). No significant between-group differences in pain (VAS) or quadriceps strength. Indicates TENS may improve stair climbing ability in early KOA by facilitating smoother movement transitions.
Klika et al. (45)	RCT	64.2 ± 6.2	66 (44/22)	Standard rehabilitation after TKA	Home-based NMES (CyMedica e-vive system), garment-integrated with 3 electrodes on quadriceps (VMO, rectus femoris), app-controlled	Monophasic, 50 pps, 5 ms pulse width, duty cycle 25% (12 s contraction/10 s rest), intensity 15–85 V, 3 × 20 min/day	12 weeks (average ≥200 min/week defined as compliant)	Isometric quadriceps strength (hand-held dynamometer). Secondary: ROM, resting pain, Timed Up and Go (TUG), Stair Climb Test, KOOS, VR-12 (PCS, MCS)	NMES group had significantly greater quadriceps strength gains vs controls at 3 (*p* = 0.050) and 6 weeks (*p* = 0.015). TUG improved significantly at 6 and 12 weeks (*p* = 0.018, *p* = 0.003). No between-group differences for ROM, pain, KOOS, or VR-12. Concluded NMES accelerates recovery of strength and mobility after TKA.
Reichenbach et al. (48)	RCT	48.38 ± 10.0	68 (34/34)	Placebo TENS	Active TENS	Burst, low-frequency or high-frequency TENS, individualized per symptoms. Electrodes placed medial/lateral to joint line. Placebo: identical protocol but current ramped down after 45s.	4 sessions in week 1, 3 sessions in week 2, 2 sessions in week 3. Each session up to 60 min. Total program: 3 weeks.	WOMAC pain subscale (0–10). WOMAC function, stiffness, global score; VAS pain; Hospital Anxiety and Depression Scale; Aberdeen Participation Measure; responder rates (≥30%/≥50% pain reduction); analgesic use; adverse events.	No significant difference in WOMAC pain at 3 weeks (Δ = −0.06; 95% CI −0.41–0.29; *p* = 0.74) or at 15 weeks. No relevant differences in function, stiffness, global scores, psychological outcomes, or analgesic intake. Adverse events were minor and similar between groups (10.4 vs. 10.6%).
Elsehrawy et al. (41)	RCT	64.8 ± 9.9	220(108/112)	Sham tVNS	Auricular tVNS applied to cymba conchae	25 Hz, 250 μs pulse width, intensity 0.25–2 mA (tolerated), 30 min/session, 3 × /week	30 min per session, 3 × /week, for 12 weeks (36 sessions)	Pain (VAS), PPT (knee and elbow), Central Sensitization Inventory (CSI), KOOS, physical performance (Chair Stand, TUG), neuropathic pain (PD-Q, DN4), psychological status (HADS)	Active tVNS significantly reduced VAS pain, improved PPT, reduced central sensitization (CSI), and improved KOOS function compared to sham. Also improved Chair Stand and TUG performance and reduced neuropathic pain scores. Anxiety and depression scores improved modestly. No serious adverse events, only mild skin irritation reported.

BPI, brief pain inventory; CPM, conditioned pain modulation; CRP, C-reactive protein; CSQ-8, Client Satisfaction Questionnaire; CSI, Central Sensitization Inventory; D, duration; DLPFC, dorsolateral prefrontal cortex; EA, electroacupuncture; fNIRS, functional near-infrared spectroscopy; F, frequency; FROM, flexion range of motion; HADS, Hospital Anxiety and Depression Scale; HSS, Hospital for Special Surgery score; IL-6/IL-10, interleukins 6 and 10; KOOS, Knee injury and Osteoarthritis Outcome Score; M1, primary motor cortex; MCS, mental component summary (VR-12); NMES, neuromuscular electrical stimulation; NRS, Numeric Rating Scale; PPT, pressure pain threshold; PT, physical therapy; QST, quantitative sensory testing; RCT, randomized controlled trial; ROM, range of motion; SF-36, Short Form Health Survey-36; Sham, sham stimulation (placebo); S1, primary somatosensory cortex; SO, supraorbital; tDCS, transcranial direct current stimulation; TENS, transcutaneous electrical nerve stimulation; tVNS, transcutaneous vagus nerve stimulation; TG, thigh girth; TKA, total knee arthroplasty; TUG, Timed Up and Go; US, ultrasound; VAS, Visual Analog Scale; VMO, vastus medialis oblique; WB-EMS, whole-body electromyostimulation; WOMAC, Western Ontario and McMaster Universities Osteoarthritis Index; VR-12, Veterans RAND 12-Item Health Survey; 6MWT, 6-min Walk Test.

### 3.4 Study results

This review included 15 randomized controlled trials that investigated the effects of different NIES modalities, namely tDCS, transcutaneous electrical nerve stimulation (TENS), neuromuscular electrical stimulation (NMES)/whole-body electromyostimulation (WB-EMS), and tVNS, on pain, muscle strength, and physical function in older adults with KOA. Overall, the interventions tended to reduce pain intensity, improve functional performance, and in several cases, enhance quality of life. However, effect sizes and consistency varied depending on stimulation modality, stimulation parameters, and the outcomes assessed. Pain outcomes: Pain intensity was evaluated in almost all included trials, most frequently with VAS, WOMAC pain, KOOS pain, or BPI. The majority of studies (34–41) reported statistically significant reductions in pain compared with controls, particularly with tDCS and NMES-based interventions. Anti-inflammatory effects (e.g., reductions in IL-6, IL-10, TNF-α, β-endorphin) were also observed in one study (42). In contrast, one large RCT (43) found no meaningful benefit of TENS over placebo. Functional capacity: Physical function was commonly assessed through functional tests (Timed Up and Go, chair stand, 6-min Walk Test, stair-climbing) or patient-reported scales (WOMAC, KOOS function). Significant improvements were observed in trials using NMES and WB-EMS (39, 43–45), as well as in several tDCS-based studies (34, 35, 46). These benefits were particularly notable in interventions lasting ≥4 weeks and delivered with high adherence. Muscle strength and structural outcomes: Improvements in quadriceps strength and thickness (VMO) were consistently reported in NMES studies (39, 45), supporting its role in counteracting sarcopenia and joint instability in older adults. Quality of life and secondary outcomes: Some trials also reported gains in quality of life (SF-36, VR-12) and psychological dimensions, especially when stimulation was combined with exercise or rehabilitation programs (35, 46). In general, studies with higher frequency (≥3 sessions/week), longer duration (≥4–6 weeks), and multimodal approaches (electrical stimulation + physiotherapy/exercise) were more likely to demonstrate significant improvements in pain and function. Nonetheless, the heterogeneity in stimulation parameters, control conditions, and outcome measures limits direct comparisons across studies. Taken together, these results support the potential of non-invasive electrical stimulation as a complementary therapeutic strategy for reducing pain and improving functional outcomes in older adults with KOA, although the magnitude and durability of effects vary by modality and study design.

### 3.5 Meta-analysis

**Figure 3:**
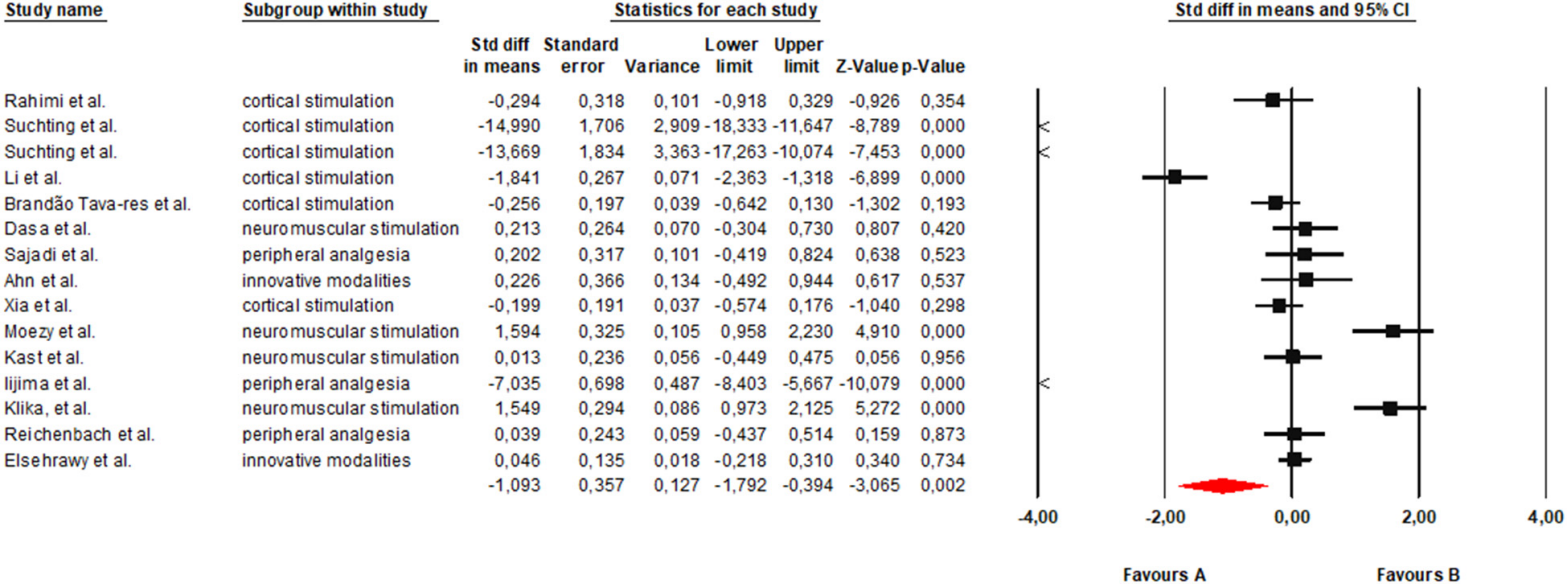
Forest plot of the global meta-analysis evaluating the overall effects of non-invasive electrical stimulation modalities in adults with KOA (random-effects model). v.

The meta-analysis included a total of 15 randomized clinical trials evaluating the effects of different non-invasive electrical stimulation modalities on clinically relevant outcomes in adults with knee osteoarthritis. Meta-analyses were conducted primarily according to the type of stimulation modality rather than by individual outcome domains. For each intervention category, pain- and/or function-related outcomes reported in the original trials were standardized and pooled as indicators of overall clinical response. When multiple eligible outcomes were reported within a study, the primary outcome identified by the authors or the most clinically relevant measure was selected to avoid double counting. Under the random-effects model, non-invasive electrical stimulation showed a statistically significant overall benefit compared with control conditions, with a moderate to large effect size (*SMD* = −1.093; 95% CI: −1.792 to −0.394; *p* = 0.002) (Figure 3). Heterogeneity was very high (*Q* = 337.05; *df* = 14; *p* < 0.001; *I*^2^ = 95.8%), indicating substantial between-study variability. This heterogeneity is likely attributable to differences in stimulation modality (e.g., tDCS, NMES/WB-EMS, TENS, CES/tVNS), stimulation parameters (intensity, frequency, and duration), participant characteristics (age, osteoarthritis severity, comorbidities), and the diversity of outcome assessment instruments used across trials (e.g., VAS, WOMAC, KOOS, and performance-based functional tests). Overall, these findings suggest that non-invasive electrical stimulation may provide clinically meaningful benefits across modalities, while also justifying the use of modality-specific subgroup analyses to better explain the observed heterogeneity.

#### 3.5.1 Subgroup analysis

##### 3.5.1.1 Cortical stimulation (tDCS)

**Figure 4:**
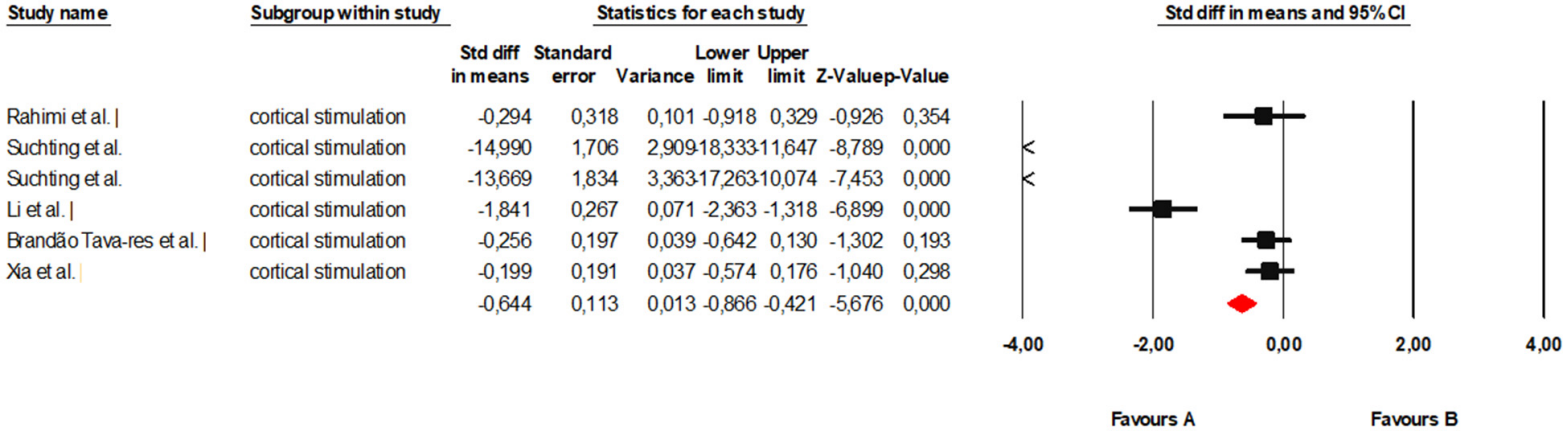
Forest plot of the global meta-analysis on the effects of cortical stimulation on pain and function in adults with KOA.

The specific analysis of studies using tDCS included six clinical trials (34, 35, 38, 42, 46, 47). The meta-analysis showed a statistically significant overall effect favoring tDCS in reducing pain and improving function compared to controls. The pooled standardized mean difference under the random-effects model was −0.64 (95% CI: −0.87 to −0.42; *p* < 0.001). This value indicates a moderate to large effect size benefiting the intervention (Figure 4).

**Figure 5:**
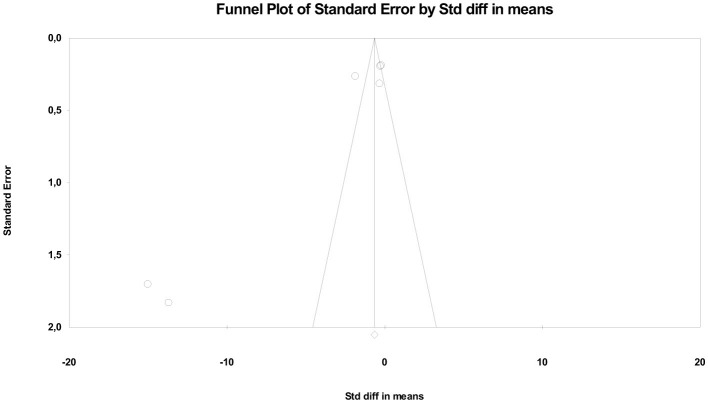
Funnel plot for cortical stimulation.

Heterogeneity was high (*Q* = 151.82; *df* = 5; *p* < 0.001; *I*^2^ ≈ 96%), reflecting significant variability across trials. This dispersion is likely due to methodological differences in stimulation protocols (current intensity, number of sessions, and electrode location), as well as the combination with other therapeutic approaches (exercise, multimodal physiotherapy, or electroacupuncture). Regarding publication bias, statistical tests provided evidence of asymmetry. Egger's regression showed a significant intercept (β = −8.71; *p* = 0.01), and the Begg and Mazumdar test suggested a tendency toward bias (tau = −0.73; *p* = 0.039). However, Duval and Tweedie's trim and fill analysis did not impute missing studies, keeping the adjusted estimate identical to the observed one (*SMD* = −0.64). The robustness analysis using the fail-safe N test indicated that more than 170 studies with a null effect would be needed to invalidate the significance of the finding, supporting the stability of the results (Figure 5). In summary, the pooled evidence suggests that tDCS represents an effective cortical stimulation modality for improving pain and function in patients with KOA, although high heterogeneity and potential publication bias limit the certainty of the estimate.

##### 3.5.1.2 Neuromuscular stimulation

**Figure 6:**
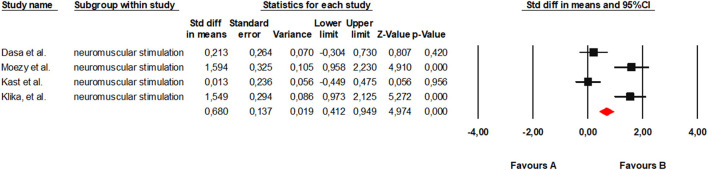
Forest plot of the global meta-analysis on the effects of neuromuscular stimulation on pain and function in adults with KOA.

Subgroup analysis for neuromuscular stimulation (including NMES and WB-EMS) included four trials (39, 40, 44, 45). Results under the random-effects model showed a significant effect in favor of the intervention (*SMD* = 0.83; 95% CI: −0.00–1.65; *p* = 0.050). Heterogeneity across studies was substantial (*Q* = 27.81; *df* = 3; *p* < 0.001; *I*^2^ = 89.2%), suggesting considerable variability among individual outcomes (Figure 6).

**Figure 7:**
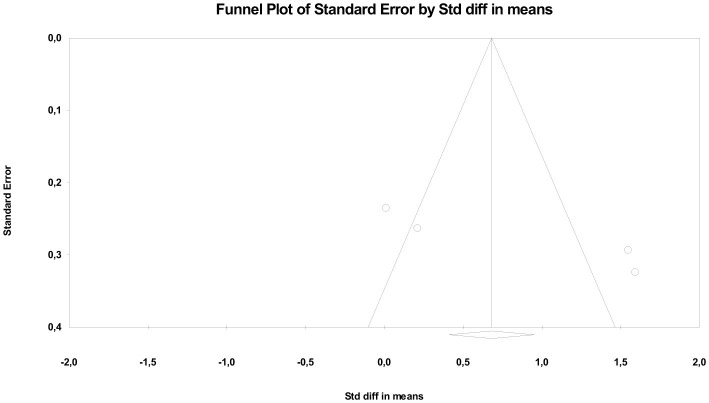
Funnel plot for neuromuscular stimulation.

Regarding publication bias, Duval and Tweedie's trim and fill analysis indicated the possible absence of a study on the left side of the mean, which slightly adjusted the combined effect towards lower values (adjusted *SMD* = 0.48). However, both Begg's test and Kendall's correlation showed no statistically significant evidence of publication bias (*tau* = 0.50; *p* = 0.308). Egger's regression suggested some asymmetry in the funnel plot (intercept = 20.65; one-sided *p* = 0.034), although this evidence should be interpreted with caution given the small number of included studies (Figure 7).

##### 3.5.1.3 Peripheral analgesia (TENS)

**Figure 8:**
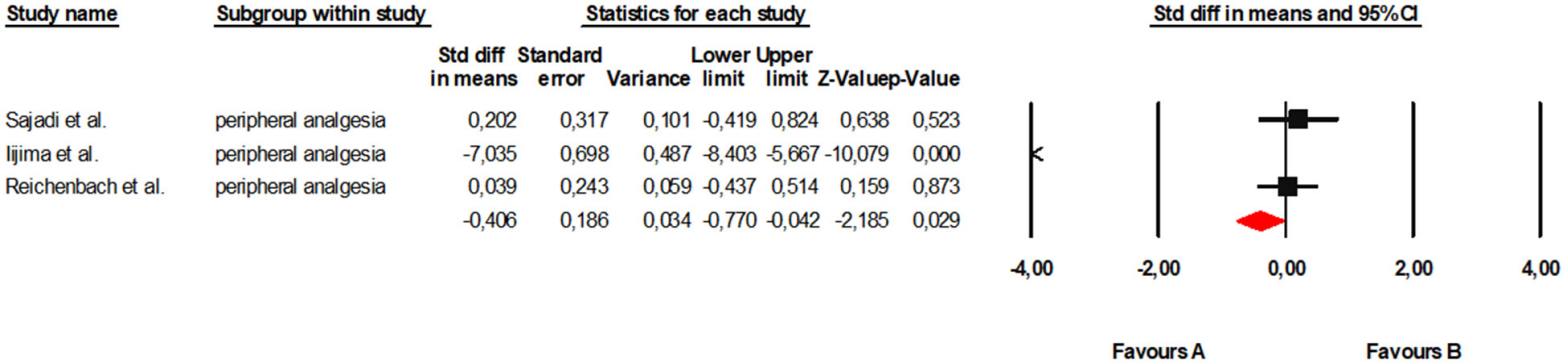
Forest plot of the global meta-analysis on the effects of peripheral analgesia on pain and function in adults with KOA.

Subgroup analysis for TENS included three clinical trials (36, 43, 48). Under the random-effects model, a small but statistically significant effect against the intervention was observed (SMD = −0.41; 95% CI: −0.77 to −0.04; *p* = 0.029). Heterogeneity was very high (*Q* = 97.23; *df* = 2; *p* < 0.001; *I*^2^ = 97.9%), indicating considerable inconsistency across studies (Figure 8).

**Figure 9:**
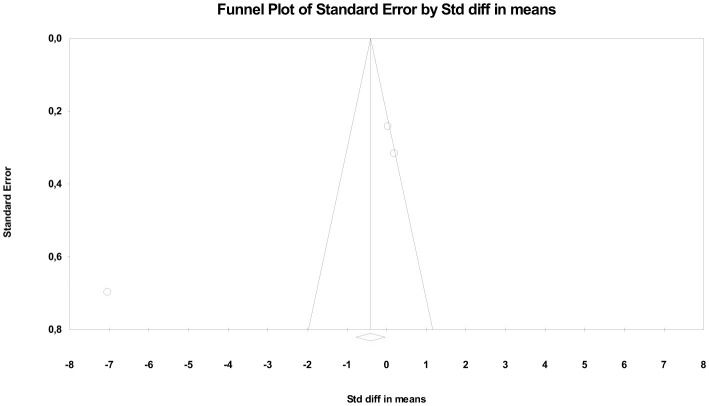
Funnel plot for peripheral analgesia.

Regarding publication bias, Duval and Tweedie's trim and fill analysis did not suggest any missing studies, leaving the effect size virtually unchanged. Begg's test and Kendall's correlation did not reach statistical significance (tau = −0.66; *p* = 0.296), and Egger's regression also showed no clear evidence of asymmetry (intercept = −14.96; *p* = 0.221). However, given the low number of included studies, these tests lack sufficient statistical power to exclude the possibility of bias. Visual inspection of the funnel plot showed some dispersion, likely attributable to the marked heterogeneity (Figure 9). Overall, the results for TENS show high variability between studies and a lack of consistency in the observed effects, limiting the strength of the available evidence for this modality.

##### 3.5.1.4 Innovative modalities

Within the category of innovative stimulation modalities, only two clinical trials were identified: Ahn et al. (37), who evaluated an emerging electrical stimulation technique designed to modulate cortical activity and improve functional recovery after surgery, and Elsehrawy et al. (41), who proposed a hybrid approach with experimental features aimed at optimizing both pain control and postoperative mobility. The small number of studies (*n* = 2) did not allow for a formal meta-analysis, given that quantitative synthesis methods require a minimum of three trials to obtain valid pooled estimates. However, a qualitative review of these studies provides relevant information for understanding the potential of these emerging techniques. Both studies showed encouraging preliminary results, with a trend toward reduction in pain intensity and improvement in functional parameters, although the magnitude of the effect and the consistency of the findings were heterogeneous. It is important to highlight that innovative modalities represent an expanding field of research, the value of which lies in offering complementary alternatives to traditional stimulation techniques. However, the evidence available to date is insufficient to draw definitive conclusions, due not only to the small number of trials, but also to the methodological limitations inherent in exploratory studies (small samples, pilot designs, and variability in stimulation protocols).

## 4 Discussion

The objective of this study was to synthesize the available evidence on the effects of NIES on pain and function in middle-aged and older adults with KOA. Overall, the findings of this review allow a direct response to the research questions posed. First, regarding the effects of non-invasive electrical stimulation on pain and physical function, the available evidence indicates that most modalities are associated with clinically relevant reductions in pain and, to a lesser extent, improvements in functional outcomes in middle-aged and older adults with KOA, particularly when interventions are delivered over longer periods and with adequate adherence. Second, when comparing different stimulation modalities, cortical stimulation using transcranial direct current stimulation showed the most consistent analgesic effects, whereas neuromuscular stimulation demonstrated clearer benefits for muscle strength and functional mobility, especially in individuals with quadriceps weakness; in contrast, the effects of transcutaneous electrical nerve stimulation were highly variable and less robust. These differences between stimulation modalities may be partially explained by their distinct physiological mechanisms. Techniques targeting cortical neuromodulation (e.g., tDCS or CES) primarily influence central pain processing and descending inhibitory pathways, whereas neuromuscular stimulation modalities mainly act through peripheral motor activation and improvements in muscle function. Peripheral sensory stimulation techniques such as TENS, in turn, mainly provide short-term analgesic modulation through sensory gating mechanisms. Finally, with respect to factors associated with greater effectiveness, the results suggest that intervention-related characteristics such as stimulation modality, number of sessions, duration of treatment, and combination with exercise or physiotherapy, as well as participant-related factors such as baseline muscle weakness, appear to influence the magnitude of pain reduction and functional improvement. The results show that, although the magnitude and consistency of effects vary by modality, most trials suggest clinically relevant benefits in terms of pain reduction and functional improvement, especially when interventions are applied over a long period of time and with high adherence. These findings reinforce the potential of NIES as a complementary strategy to conventional interventions, with the advantage of being well tolerated and safe in a population characterized by comorbidities and a higher risk of adverse drug effects. From a clinical perspective, the magnitude of the effects observed across the different non-invasive electrical stimulation modalities appears potentially meaningful. Trials evaluating cortical stimulation, particularly tDCS, typically reported reductions in pain intensity in the range of approximately 1–3 points on the Visual Analogue Scale, whereas neuromuscular stimulation modalities were more often associated with modest but relevant improvements in functional scales such as WOMAC or KOOS, reflecting gains in mobility and daily activities. Although these effects do not uniformly exceed established minimal clinically important differences across all studies, they may be sufficient to facilitate greater participation in exercise-based rehabilitation and daily activities, particularly in older adults with limited tolerance to conventional interventions.

tDCS has emerged as one of the most promising non-invasive electrical stimulation modalities for knee osteoarthritis. In the studies included in this review, tDCS consistently showed a beneficial effect on pain, with moderate effect sizes, while results on physical function were more heterogeneous. For example, Rahimi et al. (34) and Brandão Tavares et al. (35) reported significant improvements in pain, functional performance, and quality of life, especially when tDCS was applied to the primary motor cortex (M1). In contrast, studies by Suchting et al. (42, 47) showed clearer effects on inflammatory biomarkers and experimental pain sensitivity, but with a less marked clinical impact on function. Similarly, Xia et al. (38) showed that the combination of tDCS with TENS improved gait and pain parameters, although the benefits tended to fade at follow-up beyond the first month. The analgesic effects associated with tDCS are biologically plausible and can be explained by its capacity to modulate cortical excitability and enhance descending inhibitory pain pathways. Stimulation over the primary motor cortex has been shown to influence central pain processing, reduce central sensitization, and improve conditioned pain modulation, mechanisms that are particularly relevant in chronic knee osteoarthritis. These central effects may account for the more consistent reductions in pain observed with tDCS compared with its more variable impact on functional outcomes, which are likely influenced by additional peripheral and musculoskeletal factors. These findings are consistent with recent reviews and meta-analyses. A systematic synthesis by Yang et al. (49) demonstrated that tDCS is associated with a significant reduction in pain in knee osteoarthritis, although without consistent improvements in joint function or stiffness. Similarly, Wu et al. (50) confirmed a short-term positive effect on pain but no sustained benefit in functional parameters. Likewise, a broader meta-analysis by Dai and Liu (51) indicated that protocols with a higher number of sessions (≥10) and stimulation over M1 were associated with greater reductions in pain, suggesting a possible dose–response effect.

The results of our review indicate that neuromuscular electrical stimulation, including both localized (NMES) and whole-body (WB-EMS) modalities, is associated with clinical benefits in terms of pain, quadriceps strength, and functional mobility, particularly in populations with knee osteoarthritis and documented muscle weakness. For example, the home-based trial of app-based NMES demonstrated that within 12 weeks, patients achieved clinically relevant reductions in pain and stiffness, as well as improvements in isometric quadriceps strength (52, 53). However, when these findings are contrasted with the broader evidence, a more nuanced picture emerges. A recent systematic review including six trials (*n* ≈ 367) concluded that the addition of NMES to exercise in knee osteoarthritis did not outperform exercise alone in improving self-reported pain or function (*SMD* = −0.33; 95% CI: −1.05–0.39; *p* = 0.37) (27). This is consistent with previous analyses that rated the overall evidence for NMES in knee osteoarthritis as inconsistent or of low quality (22). The functional benefits associated with neuromuscular electrical stimulation are biologically plausible and consistent with its capacity to directly activate motor units, enhance muscle recruitment, and counteract atherogenic muscle inhibition. In older adults with knee osteoarthritis, these mechanisms may contribute to improved quadriceps strength and neuromuscular control, which are critical determinants of mobility and functional independence. Furthermore, research on WB-EMS suggests that the simultaneous contraction of multiple muscle groups and the activation of anabolic and inflammatory pathways may further contribute to functional improvement (54).

The evidence for analgesic peripheral electrical stimulation, specifically TENS, in the management of knee osteoarthritis is the most inconsistent among the interventions analyzed. A network meta-analysis including 27 trials and six types of electrical stimulation therapies concluded that the recommendation for low-frequency TENS and NMES was unclear for pain relief in knee osteoarthritis, whereas only interferential current showed a significant effect (29, 55). More recent reviews have reported mixed results. A meta-analysis of 14 trials found that TENS was associated with a reduction in pain (*SMD* = −0.79; 95% CI: −1.31–−0.27; *p* < 0.00001), but without significant improvements in WOMAC-assessed function (*SMD* = −0.13; 95% CI: −0.35–0.10; *p* = 0.09) (56). Similarly, another systematic review reported that, although TENS combined with other interventions improved gait capacity and reduced pain in knee osteoarthritis, the effects on joint stiffness were negligible. From a clinical and physiological perspective, the variable effects observed with TENS are consistent with its primary mechanism of action, which is based on sensory gating and short-term modulation of nociceptive input through the activation of inhibitory afferent pathways (gate control theory). Cutaneous stimulation of Aβ fibers can reduce nociceptive signal transmission and activate endogenous analgesic mechanisms, providing a plausible explanation for the short-term pain relief commonly reported with this intervention (56). This mechanism may be particularly relevant in patients with a predominantly nociceptive pain profile, such as those in the earlier stages of knee osteoarthritis. For example, Shimoura et al. (57) reported improvements in pain intensity and walking capacity following a single session of TENS in individuals with pre-radiographic knee osteoarthritis. Similarly, TENS may be considered a low-risk, temporary, supportive intervention rather than a stand-alone treatment. Its clinical utility appears greatest when integrated into a multimodal rehabilitation program, for example to facilitate movement or exercise by reducing pain. However, current evidence does not support its superiority over standard care or therapeutic exercise, and its use should be guided by clearly defined objectives, careful monitoring of response, and individualized adjustment of stimulation parameters.

The emerging category of electrical stimulation modalities includes interventions such as cranial direct/alternating CES and tVNS. Although the available evidence is still very limited, only two trials met the inclusion criteria in this review, the individual results offer promising signs. For example, the Feasibility and Efficacy of Remotely Supervised Cranial Electrical Stimulation for Pain in Older Adults with KOA study (37) demonstrated that ten sessions of supervised home-based CES reduced clinical pain (NRS) and increased pressure pain thresholds and conditioned pain modulation in older adults with KOA (40). The latter showed that an auricular tVNS protocol (3 sessions/week for 12 weeks) produced statistically significant improvements in the pain VAS, PPT, neuropathic questionnaires (PD-Q, DN4), and the KOOS function subscale in patients with KOA. From a pathophysiological perspective, these modalities have compelling rationales: in the case of tVNS, stimulation of the auricular branch of the vagus nerve can activate pain-inhibiting reflex pathways, modulate parasympathetic tone, and reduce central sensitization, all mechanisms implicated in KOA pain (58). For its part, CES applied to cranial regions can alter cortical activity related to pain processing, enhancing descending inhibition and reducing central hypersensitivity (59, 60). Therefore, although these studies open up a line of research with great potential, in current clinical practice its use should be considered as experimental or complementary, rather than as a first-line intervention. It is suggested that future research include randomized designs with larger sample sizes, standardized stimulation protocols, extended follow-up, evaluation of clinical and functional out-comes, and transparency regarding stimulation parameters and adherence.

Another aspect that deserves consideration is the duration and practical applicability of the observed effects. Although many trials reported significant improvements immediately after the intervention period, follow-up assessments were often limited or showed attenuation of the benefits over time. For example, several studies evaluating cortical stimulation reported reductions in pain during or shortly after the treatment phase, but the persistence of these effects beyond the first months was less consistent. Similarly, improvements associated with neuromuscular stimulation often depended on adherence to the intervention and the continuation of strengthening activities. From a clinical perspective, these findings suggest that non-invasive electrical stimulation should be considered primarily as an adjunct to multimodal rehabilitation rather than a stand-alone intervention. In practice, its effectiveness may depend on factors such as patient adherence, the feasibility of home-based use, the need for supervision in certain protocols, and its integration with therapeutic exercise programs aimed at maintaining long-term functional improvements.

From a clinical perspective, the findings of this review suggest that different NIES modalities may be particularly relevant for specific patient profiles within the heterogeneous population of individuals with knee osteoarthritis. Cortical stimulation techniques such as tDCS may be especially useful for patients with predominant pain and features of central sensitization, where modulation of central pain processing could facilitate symptom control. In contrast, neuromuscular electrical stimulation modalities, including NMES and WB-EMS, may be particularly beneficial in patients presenting with quadriceps weakness, reduced muscle activation, or functional mobility limitations, as these interventions directly target motor unit recruitment and muscle strengthening. Peripheral sensory stimulation approaches such as TENS may be most useful as a short-term adjunct to facilitate movement or exercise in patients with activity-related pain, although the evidence for sustained functional benefits remains limited. Considering the clinical heterogeneity of KOA, tailoring the choice of electrical stimulation modality according to the predominant clinical presentation may enhance the effectiveness of rehabilitation strategies. Another clinically relevant aspect concerns the safety and tolerability of non-invasive electrical stimulation modalities in middle-aged and older adults with knee osteoarthritis, many of whom present with comorbidities. Overall, the studies included in this review reported a favorable safety profile across the different stimulation techniques. No serious adverse events related to the interventions were described. The most commonly reported side effects were mild and transient, including local skin irritation at electrode sites, tingling sensations, or temporary discomfort during stimulation. These events rarely led to treatment discontinuation. Nevertheless, certain practical considerations remain important in clinical settings, such as ensuring correct electrode placement, monitoring stimulation intensity, and providing appropriate patient education when devices are used in home-based protocols. Taken together, the available evidence suggests that NIES modalities are generally safe and well tolerated when applied according to recommended protocols, although continued monitoring of safety outcomes in larger trials and longer follow-up periods remains advisable.

This systematic review and meta-analysis have several limitations that should be considered when interpreting the findings. First, there was substantial heterogeneity across the included studies regarding the non-invasive electrical stimulation modalities and stimulation protocols used, including differences in frequency, intensity, pulse duration, electrode placement, and treatment dosage. This heterogeneity limits the precision of pooled estimates and complicates direct comparisons between intervention modalities. Second, considerable variability was observed in outcome measures and follow-up duration. Studies employed different instruments to assess pain and function, and follow-up periods were generally short, which restricts conclusions regarding the sustainability of intervention-specific effects and their relevance for long-term clinical management. Third, the possibility of publication bias cannot be excluded. The review did not systematically include unpublished studies or grey literature, and although no language restrictions were applied during database searches, the available evidence is predominantly derived from studies published in English. This may have led to an overrepresentation of studies reporting positive findings. In light of these limitations, future research should prioritize the standardization and optimization of non-invasive electrical stimulation protocols for knee osteoarthritis, with clearer reporting of stimulation parameters such as intensity, frequency, pulse duration, electrode placement, and treatment dosage for each intervention modality. Well-designed randomized controlled trials with larger sample sizes and longer follow-up periods are needed to evaluate the sustainability of intervention-specific effects and their impact on long-term clinically relevant outcomes, including mobility, functional independence, and quality of life. In addition, comparative studies examining different non-invasive electrical stimulation modalities within similar clinical contexts may help to identify which interventions are most appropriate for specific patient profiles and stages of disease.

## 5 Conclusion

This systematic review and meta-analysis suggest that non-invasive electrical stimulation may represent a useful adjunctive intervention in the management of knee osteoarthritis in middle-aged and older adults, although its effectiveness varies according to the stimulation modality and clinical context. First, cortical stimulation using transcranial direct current stimulation (tDCS) demonstrated the most consistent effects on pain reduction, supporting its potential role as an adjuvant therapy, particularly when combined with exercise or conventional physiotherapy. Second, neuromuscular stimulation modalities, including NMES and WB-EMS, showed beneficial effects on quadriceps strength and functional mobility, especially in patients presenting with muscle weakness or functional limitations, although their superiority over exercise-based interventions alone remains uncertain. Third, the evidence for peripheral sensory stimulation with TENS was more heterogeneous, suggesting that this modality may be most appropriately used as a short-term complementary strategy to facilitate movement or participation in rehabilitation. Emerging approaches such as cranial electrical stimulation (CES) and transcutaneous vagus nerve stimulation (tVNS) appear promising, but the current evidence remains limited and requires confirmation in larger and methodologically robust trials. From a clinical perspective, these findings support the cautious integration of non-invasive electrical stimulation into multimodal rehabilitation programs for patients with knee osteoarthritis, particularly those with persistent pain or reduced tolerance to conventional exercise-based interventions. However, the overall certainty of the evidence should be considered moderate due to heterogeneity in stimulation protocols, variability in study quality, limited sample sizes, and the scarcity of long-term follow-up data. Future research should prioritize the standardization of stimulation protocols (e.g., intensity, frequency, electrode placement, and treatment duration), the identification of patient profiles most likely to benefit from specific modalities, and the integration of electrical stimulation with structured exercise-based rehabilitation programs, as well as longer follow-up periods and direct comparisons between modalities to better define the role of electrical stimulation within comprehensive rehabilitation strategies for knee osteoarthritis.

## Data Availability

The datasets generated and analyzed during the current study are available from the corresponding author upon reasonable request. Due to the sensitive nature of the information collected, participants were assured that their data would remain confidential and would not be publicly disclosed.
